# Use of routinely collected health data in randomised clinical trials: comparison of trial-specific death data in the BOSS trial with NHS Digital data

**DOI:** 10.1186/s13063-021-05613-x

**Published:** 2021-09-26

**Authors:** Sharon B. Love, Anna Kilanowski, Victoria Yorke-Edwards, Oliver Old, Hugh Barr, Clive Stokes, Catherine Kendall, Matthew R. Sydes

**Affiliations:** 1 MRC Clinical Trials Unit at UCL, 90 High Holborn, London, WC1V 6LJ UK; 2 Institute of Clinical Trials and Methodology, 90 High Holborn, London, WC1V 6LJ UK; 3 Institute of Epidemiology, Helmholtz Center Munich, German Research Center for Environmental Health, Ingolstädter Landstraße 1, 85764 Neuherberg, Germany; 4 Division of Metabolic and Nutritional Medicine, Dr. von Hauner Children’s Hospital, University of Munich Medical Center, Munich, Germany; 5 Gloucester Royal Hospital, Great Western Road, Gloucester, GL1 3NN UK; 6 Chestnut House, Gloucester Royal Hospital, Great Western Road, Gloucester, GL1 3NN UK; 7 Biophotonics Research Unit, Gloucestershire Royal Hospital, Great Western Road, Gloucester, GLI 3NN UK

**Keywords:** Routinely collected health data, NHS Digital, Phase III RCT

## Abstract

**Background:**

A promising approach to reduce the increasing costs of clinical trials is the use of routinely collected health data as participant data. However, the quality of this data could limit its usability as trial participant data.

**Methods:**

The BOSS trial is a randomised controlled trial comparing regular endoscopies versus endoscopies at need in patients with Barrett’s oesophagus with primary endpoint death. Data on death and cancer collected every 2 years after randomisation (trial-specific data) were compared to data received annually (all patients on one date) from the routinely collected health data source National Health Service (NHS) Digital. We investigated completeness, agreement and timeliness and looked at the implications for the primary trial outcome. Completeness and agreement were assessed by evaluating the number of reported and missing cases and any disparities between reported dates. Timeliness was considered by graphing the year a death was first reported in the trial-specific data against that for NHS Digital data. Implications on the primary trial outcome, overall survival, of using one of the data sources alone were investigated using Kaplan-Meier graphs. To assess the utility of cause of death and cancer diagnoses, oesophageal cancer cases were compared.

**Results:**

NHS Digital datasets included more deaths and often reported them sooner than the trial-specific data. The number reported as being from oesophageal cancer was similar in both datasets. Due to time lag in reporting and missing cases, the event rate appeared higher using the NHS Digital data.

**Conclusion:**

NHS Digital death data is useful for calculating overall survival where trial-specific follow-up is only every 2 years from randomisation and the follow-up requires patient response. The cancer data was not a large enough sample to assess usability. We suggest that this assessment of registry data is done for more phase III RCTs and for more registry data to get a more complete picture of when RCHD would be useful in phase III RCT.

**Trial registration:**

ISRCTN54190466 (BOSS) 1 Oct 2009.

**Supplementary Information:**

The online version contains supplementary material available at 10.1186/s13063-021-05613-x.

## Background

In the last two decades, the trend towards larger and multinational trials has shaped the landscape for high-impact clinical research [1]. With an increasing number of sites, sample sizes and trial teams to manage those, some simplification and cost reduction are needed in order to continue to run trials at the same rate [2]. A solution could be the use of routinely collected health data (RCHD) as trial data. Managed by numerous providers in the UK, National Health Service (NHS) data can be accessed for research purposes, and therefore, they have the potential to change the way of conducting randomised clinical trials (RCTs). Although discussed by several publications as a new promising technology, there is no agreed assessment of whether the quality of data is sufficient for trial use [2].

Despite the query over data quality, several RCTs have already taken advantage of RCHD in the conduct of their research. One of the first reported was the Swedish TASTE (Thrombus Aspiration in ST-Elevation myocardial infarction in Scandinavia) trial, a randomised registry-based trial using a Swedish register for identification, randomisation and follow-up of participants [3]. This approach saved more than 90% of the costs of a regular trial of comparable size, without any participants lost to follow-up [4]. In the UK, the SIMPLIFIED trial in urology is relying solely on RCHD data [5].

Reports of evaluations of data utility, as was done for the Finnish [6, 7] and the Norwegian [8] cancer registers, are limited for NHS data. We need multiple comparisons for each type of data before RCHD can be exploited for all UK trials.

The aim of this study is to assess whether RCHD in the UK might be used as participant data in RCTs, accounting for the reporting cycles agreed for each data source. For this, RCHD from NHS Digital was compared to regular trial-specific data from the phase III RCT Barrett’s Oesophagus Surveillance versus Endoscopy at Need Study (BOSS) ISRCTN54190466. This case study will investigate the usability of death and cancer data in a UK-based RCT and could therefore serve as an indication of the possibilities.

## Methods

### Design and setting

This was a retrospectively designed embedded Study Within A Trial [9] (or SWAT) assessing two sources of prospectively collected data. This study compares death and cancer in trial-specific data from the UK-based BOSS trial to the same outcomes provided by NHS Digital. The completeness, agreement and timeliness of reported outcome events are investigated to assess these key aspects of data quality [10, 11]. The BOSS trial is an RCT assessing the impact of regular endoscopies in patients with Barrett’s oesophagus on overall survival (OS) [12]. The trial recruited 3453 patients between 2009 and 2012. The trial is ongoing (primary results due 2022) and permission to use the data was given by the Chief Investigator and the Data Monitoring Committee. No accumulating comparative data related to the randomised intervention in BOSS are revealed here.

### Data sources and material

Two data sources were used in this study covering 2013–2018: BOSS trial-specific data collected from sites and patients on a 2-yearly basis, and RCHD collected annually from NHS Digital. In the BOSS trial, participants are followed up every 2 years [12]. Those in the surveillance arm are sent an endoscopy appointment and those in the endoscopy-at-need arm are sent a quality-of-life questionnaire for completion. Therefore, all follow-up requires action from participants, either to attend an appointment or to send back a questionnaire. All participants were flagged in the NHS Digital original system (called ONS [13, 14]) during randomisation in order to ease data collection; records were linked using NHS number, surname, forename, sex, date of birth, last known address and last known postcode; this was completed in 2011 and the linkage rate was not stored. Registry data from NHS Digital were received annually via ONS and are used to check against trial-reported cancers and deaths. The dates of data freeze each year were approximately the end of March but were not identical for both datasets (see Additional file 1 Table A1).

Both datasets include the trial-specific patient identifier, date and cause of death; cause of death was defined by the International Classification of Diseases (ICD)-10 codes for the NHS Digital datasets (ICD-10 diagnosis C15.9 was used for oesophageal cancer) and classified from free text for trial-specific data collection datasets (text oesophageal cancer or shortening thereof [AK]). Datasets for diagnoses of new cancers included the date of diagnosis and for NHS Digital data the ICD-10-cancer-code as well. However, after Apr 2017, access to this dataset was stopped because of access issues outside of the trial between Public Health England (PHE) which collected cancer diagnoses in the NHS, Office of National Statistics and NHS Digital. Therefore, this study only compared cancer cases with a date of diagnosis before 15 Nov 2016 which was the last diagnosis date included in the accumulated NHS Digital data.

### Statistical analysis

All analyses were performed using R 3.5.3 and RStudio version 1.2.1335. Kaplan-Meier plots were drawn using STATA version 16.1. The median follow-up was calculated for each source at each year. The reporting of death data was analysed to assess the three quality indicators: completeness, described as the extent to which instances of death were reported in the RCHD and trial-specific data collection [10]; agreement, the comparison of the data from the two sources; and timeliness, to investigate whether RCHD offers up-to-date access to trial events [11].

For completeness, the number of reported deaths within each dataset was compared within each data freeze year. A look within each source across years was included to assess when previously missing death dates became available. The agreement was assessed by calculating the time difference in reported dates of death between the sources where available. The mean, median and range of time difference were reported for each of the yearly datasets. Due to there being no record of the date that the information about the date of death was received in either dataset, the timeliness was demonstrated based on the year of data freeze. The year of first reporting was also investigated in regard to the actual date of death. The general understanding that deaths are available in RCHD data within 8 weeks was checked by assessing the number of reported deaths in the 8 weeks before the respective data freeze.

The cause of death analysis was outlined comparing the number of oesophageal cancer cases mentioned as one of the listed causes in trial-specific and NHS Digital datasets from the 2018 data freeze. Disparities between the stated cause of death in both datasets were identified.

The implications of using only one data source in future studies for the primary outcome of OS were assessed by performing a time-to-event analysis. To give a visual representation of the data, Kaplan-Meier graphs were created for each year from each source regardless of allocated treatment. With the NHS Digital dataset, participants not reported as dead were censored at the data freeze date of the respective year and dataset or on the date they left the NHS according to the dataset. This followed advise when we first received the NHS Digital data in 2013 (note NHS Digital then called MRIS). For the trial data, those without a death date were censored at the date of the data freeze.

In the cancer analysis, the number of reported oesophageal cancer cases up to 15 Nov 2016 was compared between the two datasets. Disparities were analysed regarding missing cases and differing dates of diagnosis. The time to oesophageal cancer diagnosis as a secondary outcome measure in BOSS [12] was also assessed. Due to the low number of cases, instead of a time-to-event analysis, the mean and median time from randomisation to diagnosis and time range were investigated, to see if the reported cases differ structurally.

A severe restriction of the BOSS trial data occurred in the 2015 data freeze. The death CRF was updated in early 2015 and a new form created. Due to an error, the death data from this new CRF was not downloaded for the 2015 data freeze of the trial-specific data collection. This was not detected by the trial statisticians until the following year, and therefore, the interpretation of results for 2015 has to be mostly excluded (personal communication).

## Results

**Fig. 1:**
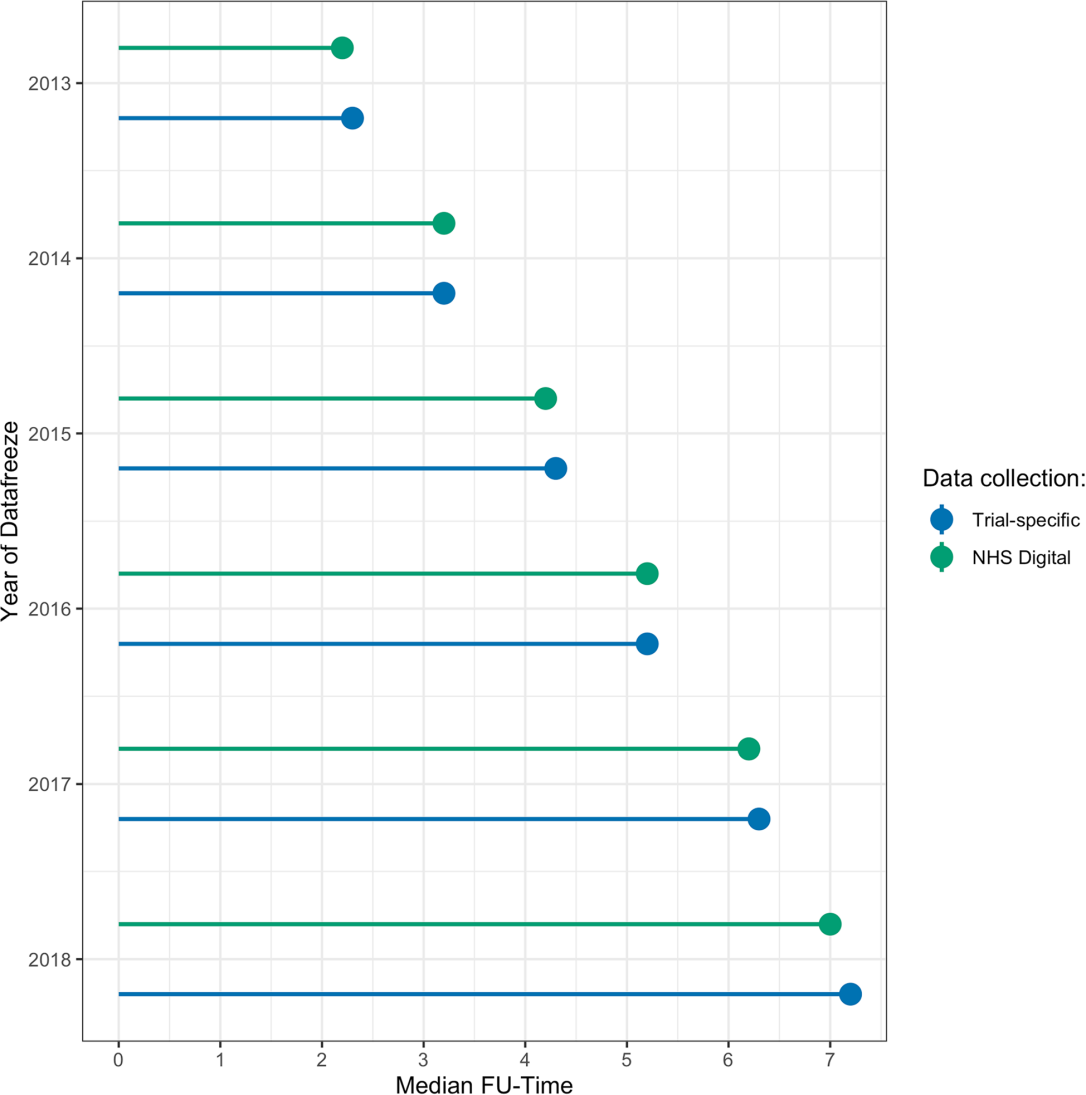
Median follow-up time in years

Median follow-up was similar between the data sources at each year (Fig. 1).

### Completeness of death reporting

**Fig. 2:**
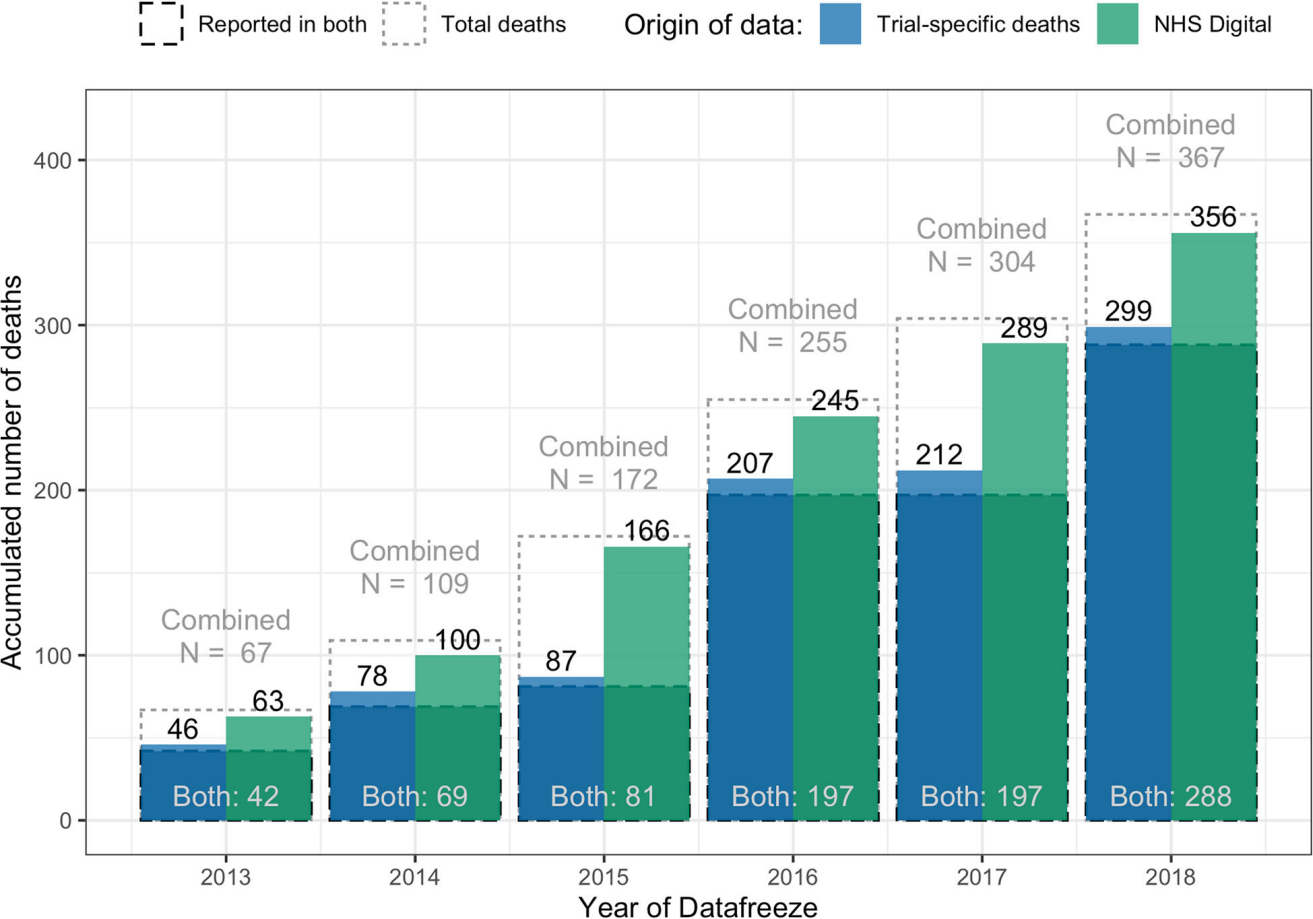
Number of deaths reported by different data sources. In this figure, deaths are accumulated over the years with colour indicating different data sources. Most deaths are reported by both sources

The majority of reported deaths at each data freeze timepoint were reported from both data sources; some deaths were only covered by trial-specific data collection and more were only reported from the NHS Digital dataset (Fig. 2).

The numbers of deaths occurring in only one dataset and the year in which the death appeared in the dataset are shown in Table 1. With the assumption that deaths from 2013 and 2014 will not be found after 2018, for either data source, only a few are persistently missing from the second data source, with 5 deaths remaining only in trial-specific data and 8 in NHS Digital data.

**Table 1:** For each data source, the numbers of deaths in each year only known from that data source, including the year at which the death was known

**a: Deaths available only in trial-specific data collection**							
**Year of event**	**Year of freeze**						
	**2013**	**2014**	**2015**	**2016**	**2017**	2018*	**Total**
**2013**	**4**	3	2	2	2	2	**15**
**2014**	–	**6**	4	3	3	3	**19**
**2015**	–	–	**0**	0	0	0	**0**
**2016**	–	–	–	**6**	5	3	**14**
**2017**	–	–	–	–	**5**	0	**5**
**2018**	–	–	–	–	–	**3**	**24**
**Total**	**4**	**9**	**6**	**11**	**15**	**11**	**56**
**b: Deaths available only in the NHS Digital dataset**							
**Year of event**	**Year of freeze**						
	**2013**	**2014**	**2015**	**2016**	**2017**	**2018**	**Total**
**2013**	**21**	9	5	4	4	4	**47**
**2014**		**22**	18	4	4	4	**52**
**2015**	–	–	**62**	16	16	12	**106**
**2016**	–	–	–	**23**	11	6	**40**
**2017**	–	–	–	–	**57**	15	**72**
**2018**	–	–	–	–	–	**27**	**212**
**Total**	**21**	**31**	**85**	**47**	**92**	**68**	**344**

The total number of deaths only in one data source in each year is given in the total column. The columns to the left are a breakdown of the total. They show when the death first appeared in the dataset

*For example, in 2018, of the 11 deaths only available in the trial-specific data collection (Table 1), two were known of in 2013, three in 2014, three in 2016 and three new in 2018. With the assumption that deaths from 2013 and 2014 will not be found after 2018, two plus three deaths result in five deaths only in trial-specific data and completely missing from NHS Digital data

### Agreement in death reporting

In 2013 and 2014, the median time difference between reported death dates in the trial and NHS Digital data is 10 days, ranging from 1 day to nearly a year (Additional file 2 Table A2). The disparities appear to vanish in 2016 and 2017 (Additional file 2 Table A2) because a new policy was employed by the trial team to simply copy the date of death given by NHS Digital data into the BOSS dataset. By 2018, the original trial date of death was favoured (Additional file 2 Table A2).

### Timeliness of death reporting

**Fig. 3:**
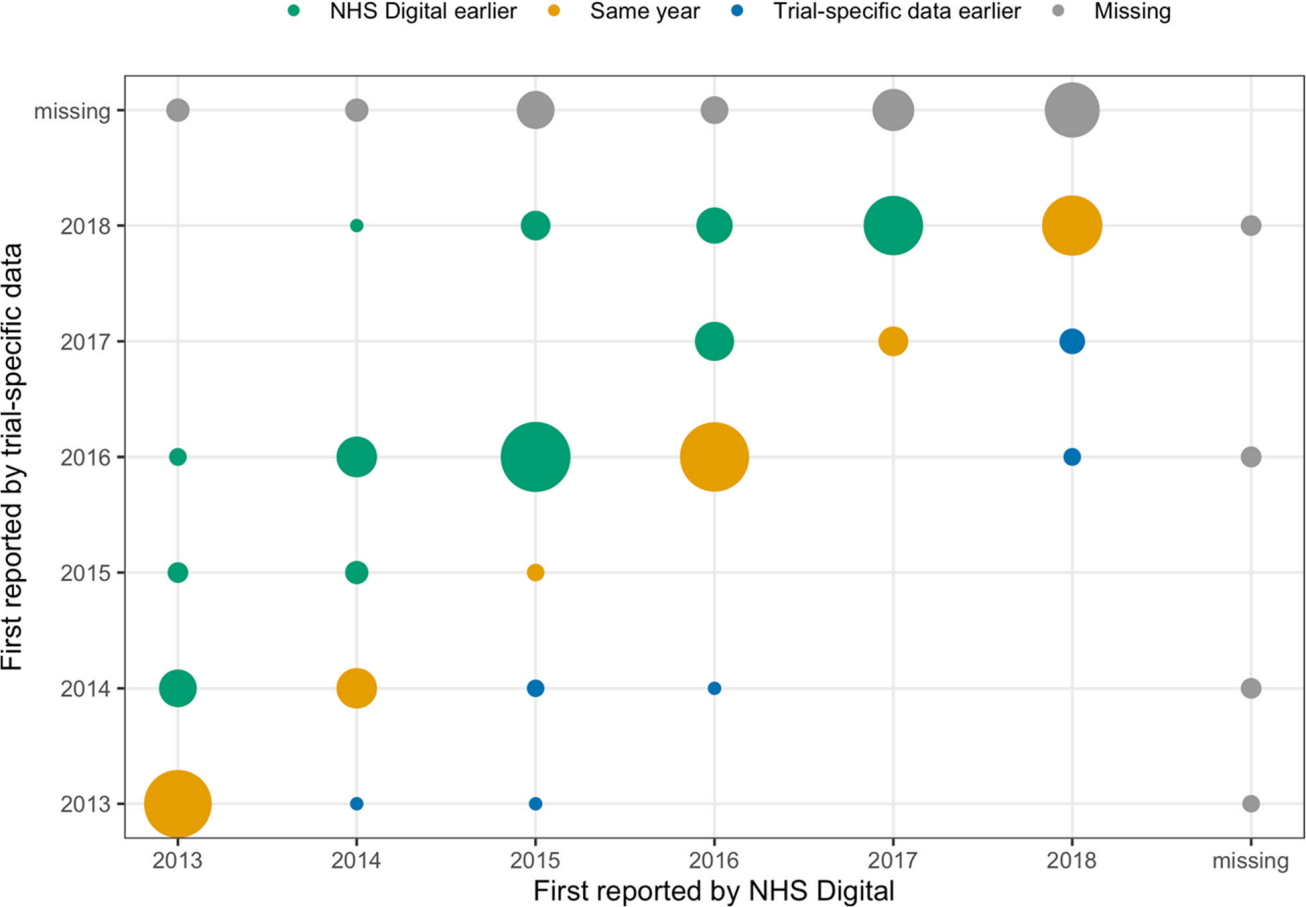
Comparison of which data source provides death information sooner. The figure depicts the year of first reporting of a death within the datasets with the size of the bubbles representing the number of deaths included in one point. Deaths reported by both datasets in the same year lie on the diagonal, those reported earlier by BOSS beyond and those reported earlier by NHS Digital above the diagonal. Missing deaths, which are reported only by one dataset, are shown in grey on the side lines

Figure 3 depicts the year of first reporting a death in the BOSS trial-specific data against the year of first reporting in NHS Digital data and therefore visualises the time lag in reporting between the two datasets. Many deaths are reported in the same year by both data sources. The remainder are mainly reported earlier by NHS Digital. In 2015, the small number of same-year-reported deaths is due to the trial-specific data extraction error, discovered by the statisticians in 2016.

**Fig. 4:**
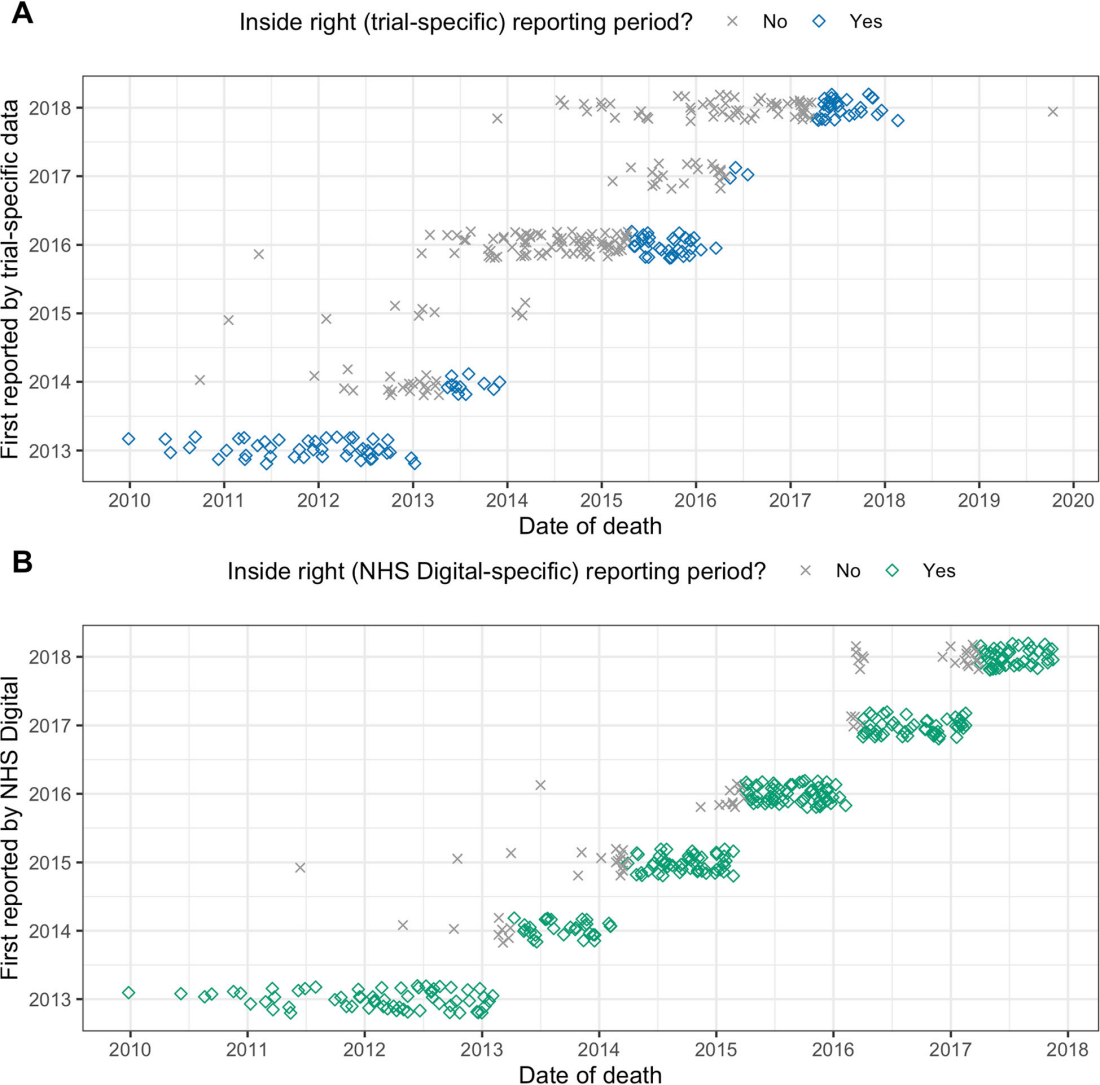
Time lag in death reporting by dataset. The time period of reporting a death is plotted against the year of death. With respect to the different data freezes, deaths were assigned to a right reporting period when the date of death actually lay between two dates of relevant data freezes. Deaths not within this period are marked as not lying inside the right reporting period

The time lag between the date of death and data freeze in which the death information appears is presented in Fig. 4. Deaths have been assigned to a respective reporting period if the death date falls within the period from one data freeze to another. This way, deaths which happen in one period but are reported in a later one are marked. This problem with time lag is more apparent in the trial-specific data collection data.

The closeness of the date of death to the data freeze was investigated for the RCHD death data (Additional file 3 Table 3). In our datasets, death dates are at least 7 weeks or more before the data freeze date.

### Agreements in the cause of death

In the 2018 data freeze, including all previous years, there were a total of 288 deaths reported by both BOSS trial data and NHS Digital (Table 2). Assessing the agreement in the reported cause of death—diagnosis, there were 282 cases (97.9%) with agreeing diagnoses. 2.1% were disagreeing causes; in two cases, the trial data reported a death due to oesophageal cancer which was not logged in NHS Digital. In four cases, NHS Digital reported oesophageal cancer as the primary cause of death which was not confirmed by trial staff.

**Table 2:** Oesophageal cancer as the cause of death within the two datasets for 2018

		BOSS trial-specific data		
		OAC	Other	Total
**NHS Digital data**	**OAC**	15	4	19
	**Other**	2	267	269
	**Total**	17	271	288

The table displays the number of deaths with oesophageal adenocarcinoma as reported cause of death in each data source. Agreements are shown, if both datasets state a death due to the diagnosis of interest or both report a death due to any other cause. *OAC* oesophageal adenocarcinoma, *Other* other non-OAC cause of death

### Implications for overall survival estimates

**Fig. 5:**
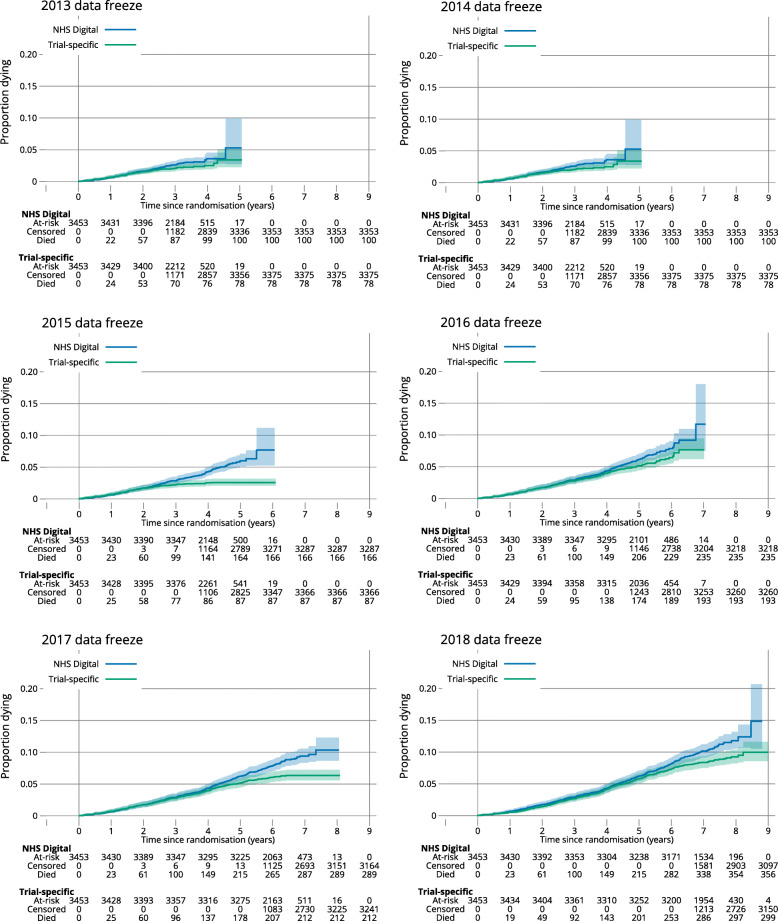
Survival probability. Kaplan-Maier plots show OS for each year divided by data source. All randomised participants were included in each plot and censored at the relevant data freeze or when they left the NHS. The gap in 2015 is partly due to the explained data extraction error

The impact on data maturity and overall survival, not split by allocated treatment group, is given in Fig. 5, depicted for every year of data freeze and separated by data source. This shows that the NHS Digital death data contains death dates for more participants across time.

### Diagnosis of new cases of oesophageal cancer

Between both datasets, there were 47 cases of oesophageal cancer reported until 15 Nov 2016. Five were only reported in the NHS Digital data, 34 only in the trial-specific data and the remaining 8 were reported in both datasets

Of the eight shared cases, none has the same date of diagnosis given in both datasets. The time difference ranges between 2 and 28 days and therefore suggests different information sources for both datasets.

Due to the limited number of cases and the differences in the datasets, no time-to-event analysis was conducted.

## Discussion

This study provides an opportunity to assess the usability of RCHD data in an RCT in the UK. Data from the BOSS trial as an ongoing UK-based trial and from NHS Digital as an important provider of information from the NHS helped to investigate the impact of data source on completeness, agreement, timeliness and trial outcomes. We assessed both death and cancer diagnosis.

The completeness and timeliness of death RCHD received annually from NHS Digital are favourable compared to the BOSS trial, which uses 2-yearly patient-actioned follow-up. Though not registry wide as in other studies [6–8], this gives some empirical evidence that UK RCHD is useful for some data in some situations. Two publications report comparisons of trial-collected and UK registry death data and found the 99% [15] and 98% [16] of deaths to be reported in both sources.

Using Table 2, we assumed that deaths first reported by one dataset in 2013 or 2014 are not going to be reported in the other dataset after a 4–5-year gap. With this assumption, a similar number of deaths were not recorded by each dataset: five cases by NHS digital and 8 by the trial-specific data. These results suggest that NHS Digital data is an efficient way of conducting follow-up in long-term RCTs.

Our consideration of agreement, cause of death and cancer analysis were not conclusive due to lack of data. The consideration of agreement between the datasets was disrupted by the trial policy in 2016 and 2017 meaning the NHS Digital death dates were accepted as the accurate data into the dataset. For the cause of death, an agreement between the datasets in the diagnosis of oesophageal cancer is limited as the numbers are small. Results from the cancer analysis are also based on small numbers, but no big difference is seen. Based on the available data, we cannot say, whether the differing number of reported cancer cases is due to the temporary access issues which started in April 2017 or whether there is room for improvement in UK cancer data. Generally, BOSS trial-specific data included more cancer cases than NHS Digital data. Due to the underlying illness required to enter the BOSS trial, participants have an increased risk of oesophageal cancer. Therefore, these patients are sensitised to this cancer and are more likely to report it to the trial team. Also, the diagnosis is more likely to happen within the same area in the hospital as the trial takes place and therefore the information is more likely to be given to the trial team.

The whole study is set within the NHS and can only present results for the RCHD used. The dates of data freeze differing a little between sources are unfortunate for our methodology study, although this should not change the conclusions since we have the information from a number of timepoints across 6 years (Additional file 1 Table A1). Another limitation in regard to assessing timeliness of reporting is that the data freezes were used as an approximation since the date of reporting was not available for either source. With a given date of reporting in addition to the actual date of death, the timeliness could have been assessed in much more detail, as it has been done in Finland [7]. We found death dates to be a minimum of 7 weeks before the data freeze date for NHS Digital data (Additional file 3 Table A3). Another limitation is the trial-specific death data being incomplete in 2015. The cause of death analysis was limited by the fact that the trial data did not use the ICD-10 codes necessary for full coding and analysis.

This methodological project was carried out on retrospectively stored data. Had it been pre-planned, we would have used the same data freeze dates for RCHD and trial data, we would have kept trial data uncorrupted from the knowledge of the RCHD, we would have requested RCHD at the same frequency as trial follow-up and we would have collected ICD-10 codes for causes of death within the trial. The change in the last few months to be able to obtain an informal earlier death notification from RCHD would also be interesting to investigate. In this trial, the RCHD was received yearly. This was a balance of cost and noting that the Data Monitoring Committee met every year and would benefit from having the most up-to-date data. The RCHD could be received more frequently and the benefits of doing so warrant investigation.

Future research is needed to investigate death data from other trials, cancer and cause of death data and other types of RCT data which could be replaced by RCHD. Across many trials, we would build a picture of when specific RCHD could be used for the trial outcome. Trials could prospectively store a trial data download on the day the RCHD is received to allow a comparison. We have placed a protocol for the analysis required in the Northern Ireland Hub for Trials Methodology Research SWAT repository store (SWAT 125: Comparison of trial-collected and routinely collected death data) [17]. Our findings from the analysis of death data could influence the way long-term follow-up is conducted in RCTs, especially in trials with periods between trial appointments as long as the 2 years in the BOSS trial. This look at one trial and the death data is a start of guidelines that could steer trialists towards evidence-based understanding of when it is appropriate to use RCHD, as shown in Table A4.

## Conclusion

Successful replacement of regular long-term follow-up of participants’ survival with UK registry data is possible for some trials. The registry data from NHS Digital in this case study acquired more and earlier death data than the trial data. We conclude that NHS Digital death data is useful for calculating overall survival where trial-specific follow-up is only every 2 years from randomisation and the follow-up requires patient response. We suggest that this assessment of registry data is done for more phase III RCTs and for more types of registry data to get a fuller picture of when RCHD would be useful in phase III RCT.

## Supplementary Information

**Additional file 1: Table A1.** Dates of data freeze dd-mmm-yyyy. Description: Table of dates of the data freeze for each data source.

**Additional file 2: Table A2.** Disparities in dates of death between the datasets where death is reported from both sources. Description: Information on the differences between the dates of death in the two sources.

**Additional file 3: Table A3.** Check of death reporting prior data freeze. Description: Information on how close to the data freeze date deaths are reported.

**Additional file 4: Table A4.** Guideline table for use of RCHD in the UK. Description: A possible table for the recording of comparisons of trial and RCHD.

## Data Availability

The datasets used in this study are not publicly available due to the still ongoing status of the BOSS trial on the one hand and the ownership of data by NHSD on the other hand. Restrictions for NHS Digital data apply to the availability of data which was used under license for the BOSS trial.

## Abbreviations

BOSS: Barrett’s Oesophagus Surveillance versus Endoscopy at Need Study
RCHD: Routinely collected health data
ICD: International Classification of Diseases
NHS: National Health Service
OS: Overall survival
PHE: Public Health England
RCT: Randomised clinical trial
TASTE: Thrombus Aspiration in ST-Elevation myocardial infarction in Scandinavia
UK: United Kingdom
