# Supplementary material for: Use of routinely collected health data in randomised clinical trials: comparison of trial-specific death data in the BOSS trial with NHS Digital data
Source: Trials. 2021 Sep 26;22:654. doi: 10.1186/s13063-021-05613-x (PMC8474902; doi:10.1186/s13063-021-05613-x)
Supplement: Supplementary file 1 — Additional file 1: Table A1. Dates of data freeze dd-mmm-yyyy. Description: Table of dates of the data freeze for each data source. [file 13063_2021_5613_MOESM1_ESM.docx]

**Table A.1: Dates of data freeze dd-mmm-yyyy**

|  | *2013* | *2014* | *2015* | *2016* | *2017* | *2018* |
| --- | --- | --- | --- | --- | --- | --- |
| **BOSS** | 11-Apr-2013 | 04-Apr-2014 | 13-Apr-2015 | 03-May-2016 | 11-Apr-2017 | 13-Mar-2018 |
| **NHS Digital** | 31-Mar-2013 | 31-Mar-2014 | 31-Mar-2015 | 31-Mar-2016 | 31-Mar-2017 | 31-Dec-2017 |
