# Supplementary material for: Use of routinely collected health data in randomised clinical trials: comparison of trial-specific death data in the BOSS trial with NHS Digital data
Source: Trials. 2021 Sep 26;22:654. doi: 10.1186/s13063-021-05613-x (PMC8474902; doi:10.1186/s13063-021-05613-x)
Supplement: Supplementary file 2 — Additional file 2: Table A2. Disparities in dates of death between the datasets where death is reported from both sources. Description: Information on the differences between the dates of death in the two sources. [file 13063_2021_5613_MOESM2_ESM.docx]

**Table A2: Disparities in dates of death between the datasets where death is reported from both sources**

| **Year** | **Number of deaths available** | **Number of differing dates** | **from previous years** | | | | **Mean [days]** | **Median [days]** | **Min [days]** | **Max [days]** |
| --- | --- | --- | --- | --- | --- | --- | --- | --- | --- | --- |
|  | Both sources |  | 2013 | 2014 | 2015 | new |  |  |  |  |
| 2013 | 42 | 8 | - | - | - | 8 | 57.8 | 10 | 1 | 187 |
| 2014 | 69 | 11 | 7 | - | - | 4 | 79.1 | 10 | 1 | 326 |
| 2015 | 81 | 16 | 7 | 2 | - | 7 | 112 | 37.5 | 1 | 844 |
| 2016 | 197 | 0 | 0 | 0 | 0 | 0 | - | - | - | - |
| 2017 | 197 | 0 | 0 | 0 | 0 | 0 | - | - | - | - |
| 2018 | 288 | 77 | 7 | 2 | 6 | 62 | 171.1 | 82 | 1 | 1095 |

This table depicts the number of differing reported dates of deaths, whether these are persistent over the years as well as mean, median and range of the time difference between reported dates in BOSS and NHSD data.
