# Supplementary material for: Use of routinely collected health data in randomised clinical trials: comparison of trial-specific death data in the BOSS trial with NHS Digital data
Source: Trials. 2021 Sep 26;22:654. doi: 10.1186/s13063-021-05613-x (PMC8474902; doi:10.1186/s13063-021-05613-x)
Supplement: Supplementary file 3 — Additional file 3: Table A3. Check of death reporting prior data freeze. Description: Information on how close to the data freeze date deaths are reported. [file 13063_2021_5613_MOESM3_ESM.docx]

**Table A.3: Check of death reporting prior data freeze**

|  | **2016** | | **2017** | | **2018** | |
| --- | --- | --- | --- | --- | --- | --- |
| **Time before data feeeze in weeks** | **NHS Digital** | **Trial-specific** | **NHS Digital** | **Trial-specific** | **NHS Digital** | **Trial-specific** |
| **1** | 0 | 0 | 0 | 0 | 0 | 1* |
| **2** | 0 | 0 | 0 | 0 | 0 | 1 |
| **3** | 0 | 1 | 0 | 0 | 0 | 2** |
| **4** | 0 | 1 | 0 | 0 | 0 | 2 |
| **5** | 0 | 1 | 0 | 0 | 0 | 2 |
| **6** | 0 | 1 | 0 | 0 | 0 | 2 |
| **7** | 0 | 1 | 2 | 0 | 2 | 2 |
| **8** | 1 | 2 | 5 | 0 | 2 | 2 |
| **12** | 3 | 3 | 11 | 0 | 7 | 3 |
| **16** | 11 | 7 | 12 | 0 | 11 | 4 |
| **24** | 24 | 15 | 23 | 0 | 20 | 9 |
| **total in year** | 75 | 110 | 54 | 25 | 67 | 87 |

For this table the number of deaths reported in the weeks prior to the data freezes in 2016, 2017 and 2018 was checked. The first row therefore shows if a death was reported within the last week prior to data freeze in one of the two datasets. That is usually zero as death cases need some time to be reported in the system or to the trial team. The eighth row gives the number of deaths reported up until week 8. *Date of death due to error as displayed in figure 3. **One of these death is unknown to NHS Digital data.
