# Supplementary material for: Use of routinely collected health data in randomised clinical trials: comparison of trial-specific death data in the BOSS trial with NHS Digital data
Source: Trials. 2021 Sep 26;22:654. doi: 10.1186/s13063-021-05613-x (PMC8474902; doi:10.1186/s13063-021-05613-x)
Supplement: Supplementary file 4 — Additional file 4: Table A4. Guideline table for use of RCHD in the UK. Description: A possible table for the recording of comparisons of trial and RCHD. [file 13063_2021_5613_MOESM4_ESM.docx]

Table A.4: Guideline table for use of RCHD in the UK

| **RCHD** | **Potential trial data** | **Guidance** |
| --- | --- | --- |
| Death | 2 yearly follow-up with participant action required | Use RCHD from NHS Digital |
| ….. | …. | …. |

Legend: So far only 1 row completed
